# Effects of coffee intake on skeletal muscle microvascular reactivity at rest and oxygen extraction during exercise: a randomized cross-over trial

**DOI:** 10.1080/15502783.2024.2409673

**Published:** 2024-10-01

**Authors:** Bin Leng, Haizhen Huang, Chuan Zhang

**Affiliations:** Central China Normal University, School of Physical Education and Sport, Wuhan, Hubei, China

**Keywords:** Caffeine, exercise performance, microvascular function, near-infrared spectroscopy

## Abstract

**Purpose:**

The effects of coffee ingestion on skeletal muscle microvascular function are not well understood. This study aimed to investigate the acute effects of coffee intake with varying levels of caffeine on skeletal muscle microvascular reactivity at rest and oxygen extraction during maximal incremental exercise in physically active individuals.

**Methods:**

Twenty healthy young male participants were administered coffee with low caffeine (3 mg/kg body weight; LC), high caffeine (6 mg/kg body weight; HC), and placebo (decaf) in different sessions. Skeletal muscle reactivity indexes, including tissue saturation index 10s slope (TSI10) and TSI half time recovery (TSI ½) following 5-minute ischemia were measured at rest and were measured at baseline and post-coffee consumption using near-infrared spectroscopy (NIRS). Post-coffee intake, NIRS was also used to measure microvascular oxygen extraction during exercise via maximal incremental exercise. Peak oxygen consumption and peak power output (W_peak_) were simultaneously evaluated.

**Results:**

Post-coffee consumption, TSI10 was significantly higher in the LC condition compared to placebo (*p* = 0.001) and significantly higher in the HC condition compared to placebo (*p* < 0.001). However, no difference was detected between LC and HC conditions (*p* = 0.527). HC condition also showed significant less TSI ½ compared to placebo (*p* = 0.005). However, no difference was detected for microvascular oxygen extraction during exercise, despite the greater W_peak_ found for HC condition (*p* < 0.001) compared to placebo.

**Conclusion:**

Coffee ingestion with high caffeine level (6 mg/kg body weight) significantly enhanced skeletal muscle reactivity at rest. However, the improvement of exercise performance with coffee intake is not accompanied by alterations in muscle oxygen extraction.

## 1. Introduction

Complicated relationships exist between dietary components and exercise capacity, particularly in relation to ergogenic aids and their potential to enhance athletic performance. Coffee is one of the most commonly used dietary supplements for such purpose, which contains various constitutes that are potentially ergogenic [1]. The use of coffee as a dietary supplement has elicited a lot of interest in sports and exercise science field since it has been suggested that these effects improve exercise performance due to its multi-facet physiological effects, encompassing neuromuscular, antioxidant, endocrine, cognitive, and metabolic benefits [1]. Among its many constitutes, caffeine is a popular chemical stimulant that has been known to affect many different physiological systems, including but not limited to the neural, cardiovascular, and muscular systems [2]. Research indicates that the primary effect of caffeine is on the central nervous system, which may lessen perceived effort and postpone the onset of exhaustion [3]. In addition, some suggest that caffeine may also affect microvascular blood flow [4], which in turn may affect oxygen transport and utilization.

The critical importance of microvascular function in muscle tissue during physical exertion is highlighted by its essential role during exercise [5]. This microvascular system dynamically modulates blood flow to meet the increased metabolic requirements of active muscles effectively [6]. This adept modulation is crucial in maintaining an adequate supply of oxygen and essential nutrients to the muscles during exercise. It also aids in quickly removing metabolic byproducts such as carbon dioxide and lactate, which build up during physical activities. The efficiency of this microvascular system is not only vital for sustaining muscle contraction and delaying fatigue, but is also key in boosting overall muscular endurance [7]. Furthermore, the microvascular network is also essential in the post-exercise recovery and adaptation [8,9]. For example, it is involved in the delivery of key substances (like amino acids) for muscle growth and repair [10], and also plays a role in reducing post-exercise oxidative stress [11]. Regular exercise training could increase capillary density and improve endothelial function, which enhances the muscle’s ability to extract and utilize oxygen, ultimately leading to better performance [12]. Taken together, it can be assumed that the microvascular system significantly influences both the acute and long-term aspects of exercise performance and muscle health.

The pharmacological effects of coffee provide an interesting area for research. Most of the existing studies have utilized flow-mediated dilation (FMD) technique to examine the influence of coffee intake on macrovascular function, with majorities studies indicating an acute effect. Research has shown that coffee may have a range of effects on vascular beds, including both vasodilation and constriction [13–15]. These effects depend on the amount of caffeine consumed within the coffee and the particular vascular location. The somewhat contradictory results raise intriguing concerns about how coffee affects the microvasculature of muscles, especially during exercise when proper blood flow control is crucial. Furthermore, little is known about how these microvascular reactions relate to overall exercise performance, especially when it comes to measurements like peak oxygen uptake (VO_2peak_) and peak power output (W_peak_).

It is clear that coffee use is common among athletes and people who exercise, and there is growing interest for investigating the effects of coffee use for optimizing exercise performance. However, how the skeletal muscle microvascular system responses to coffee intake is poorly understood. Therefore, the purpose of this study was to evaluate the effects of coffee intake on skeletal muscle microvascular reactivity at rest and oxygen extraction during exercise. This study aims to fill the knowledge gaps by examining the effects of acute coffee consumption that contains different doses of caffeine (3 mg/kg and 6 mg/kg body weight, along with a placebo), on human skeletal muscle microvascular responses at rest and during progressive cycling exercise, which is a common protocol for assessing maximal aerobic power and endurance performance. In addition, the research will investigate the effects of coffee intake on peak oxygen consumption and peak power production during exercise. Based on previous reports, we hypothesized that coffee intake would acutely impact muscle microvascular response and improve exercise performance.

## 2. Methods

### 2.1. Participants and ethical approval

This study was approved by the Institutional Review Board at our University and was conducted in accordance with the Declaration of Helsinki.

Twenty young healthy, physically active males who do not regularly drink coffee or any other caffeinated beverages participated in this study. The inclusion criteria were 1) between the ages of 18–30, free of any known cardiovascular, metabolic, or musculoskeletal disorders, 2) not having injured the lower extremity for the past year, 3) not currently taking any medication that may affect the cardiovascular or musculoskeletal system, 3) being physically active, defined as exercising for at least 3 times a week with each session lasts at least 60 min, 4) BMI ≤30 kg/m^2^. Written consent was obtained prior to any data collection. Physical activity level was assessed using the short version of the International Physical Activity Questionnaire [16]. Caffeine consumption habit was assessed with the Landrum’s Caffeine Consumption Questionnaire [17].

### 2.2. Study design

This is a single-group, repeated-measures design. The participants were instructed to not consume alcohol or perform strenuous exercise 24 h prior to each testing session, and not to consume any caffeinated beverages on the day of the testing until it was finished. Each participant visited a temperature- and humidity-controlled laboratory three times at the same time of the day, separated by at least 48 h. All participants were tested in the morning following overnight fast at the same time across three trials, except for 2 participants due to scheduling conflicts. These two participants were tested in the afternoon and at least 2 h following the last meal, and the testing time were kept the same for across the three trials. During the first testing session, the participant was familiarized with the testing equipment and procedure. The participant then laid down for at least 10 min, and baseline skeletal muscle reactivity at rest was measured using near-infrared spectroscopy (NIRS). Afterward, the participant drank a cup of coffee that contains different caffeine levels within 5 min. After consuming the coffee, the participant laid down for another 45 min before the post-coffee intake test starts. Skeletal microvascular reactivity at rest and oxygen extraction during exercise was assessed using near-infrared spectroscopy (NIRS). VO_2peak_ and W_peak_ were also assessed.

Participants drink coffee that contains different levels of caffeine (placebo, 3 mg/kg and 6 mg/kg body weight) in a randomized order. The Nescafé Decaf powder was prepared to serve as a placebo which contains very little caffeine [18], and the amount of powder used was the same as the high caffeine dose condition. For the 3 mg/kg (LC) and 6 mg/kg (HC) doses, the Nescafé Original coffee power which contains 3.4 g caffeine/100 g was used, and the amount was calculated based on each participant’s body weight to ensure they receive the correct amount of caffeine [18]. Coffee was served in a clear cup by mixing the coffee power with 300 ml warm water.

### 2.3. NIRS testing

Skeletal muscle microvascular reactivity at rest was assessed before and after the participant drank the coffee and lying down for 45 min using a continuous wavelength NIRS device (58 × 28 × 6 mm, PortaLite, Artinis Medical Systems B.V., Einsteinweg, The Netherlands) coupled with the post-occlusive reactive hyperemia (PORH) technique. The NIRS probe was placed on the rectus femoris muscle of the dominant leg at 1/3 distance between the patella and the anterior superior iliac spine using double-sided tape and self-adhesive wrap. A tourniquet that connects to a custom-built rapid inflation device was placed proximally to the probe and as high up as possible [19]. This set-up ensures a rapid tourniquet inflation and deflation for accurate assessment of microvascular reactivity. When the test starts, 1–2-min baseline data were first collected, and the tourniquet was rapidly inflated to ~250 mmHg to provide full vascular occlusion for 5 min. The tourniquet was then rapidly deflated, and the participant continued laying on the bed for 5 min. The NIRS signal was continuously monitored throughout the experiment. The tourniquet was then removed, while the NIRS probe was kept on the participant for microvascular oxygen extraction assessment during the incremental exercise.

This NIRS device has 3 light sources and 1 detector which enables three separation distances (3 cm, 3.5 cm and 4 cm), and can provide relative changes in oxygenated- and de-oxygenated hemoglobin concentration changes using the modified Beer-Lambert Law. It also provides the parameter of tissue saturation index (TSI) which measures the oxygenation saturation within the measured tissue using the spatially resolved spectroscopy (SRS) technique. Because NIRS measured hemoglobin concentration is influenced by adipose tissue thickness (ATT) [20], and that the TSI measured with the SRS method is likely much less susceptible to ATT [21], TSI was used for further hemodynamic calculations.

### 2.4. VO_2peak_ and W_peak_ testing

VO_2peak_ and W_peak_ were assessed using a cycle ergometer (EC3000, Customed, Ottobrunn, Germany), coupled with a portable gas analysis system (Cortex, MetaMax 3B, Leipzig, Germany). The participant performed 2-min warm-up with no resistance before the incremental test. The participant then cycled on the ergometer at 70 rpm starting at 40W with 40W increments (ΔW) every 2 min (T_stage_) until volitional exhaustion. The NIRS probe was placed on the rectus femoris muscle and collected the data throughout the cycling test. A research assistant used verbal encouragement to help the participant reach better performance at the end of the test. W_peak_ was calculated based on the workload at the final completed stage of the test (W_last_) and the time spent in the last incomplete stage (T_incomplete_) using the following equation [22]:

$$W_{\mathrm{peak}}\;=\;W_{\mathrm{last}}\;+\;T_{\mathrm{incomplete}}/T_{\mathrm{stage}}\;*\;\Delta \;W$$

### 2.5. NIRS data analysis

NIRS data were processed using custom-written Matlab scripts (version 2020b, The Mathworks, Natick, MA, USA). For NIRS microvascular reactivity assessment at rest, the initial 10-s rate of TSI increase immediately after the release of the tourniquet was determined to represent microvascular reactivity [19,23]. Additionally, the time it took for the TSI to reach half of the total PORH recovery (TSI ½) following tourniquet release was also evaluated [24].

For NIRS assessment during exercise, the microvascular oxygen extraction was calculated using averaged TSI values over the last 30s of each completed stage during the maximal incremental test. Because the participant may have completed different number of stages at different visits, two indexes were calculated to represent microvascular oxygen extraction. For the first index, the last stage that the participant was able to complete during all three trials was selected, and the change in TSI from the first to the last stage was calculated as ΔTSI_exercise1_. For the second index, the TSI change from the first to the last stage that the participant was able to complete for each individual trial was calculated as ΔTSI_exercise2_.

### 2.6. Statistics

Sample size was estimated using G*power software, and ANOVA with repeated within factors test. Given an alpha level of 0.05, power of 0.8 and an estimated effect size of 0.3, it was calculated that 20 participants are needed for this study.

All data were processed using SPSS 27 (IBM Corp., Armonk, NY, USA). Data were checked for normal distribution using the Shapiro–Wilk test. All data except for TSI10 were normally distributed. Therefore, further analyses for TSI10 were carried out using Friedman's test. Two-way repeated ANOVA was used to detect any changes for TSI ½, with one repeated factor being the level of caffeine content for the ingested coffee (placebo, LC and HC conditions), and another one being the time (pre- or post-coffee supplementation). One-way repeated ANOVA was used to detect any difference for skeletal muscle oxygen extraction during exercise (ΔTSI_exercise1_ and ΔTSI_exercise2_), as well as for W_peak_ and VO_2peak_. When necessary, post-hoc test was carried out with LSD adjustments. Significance level was set at 0.05.

## 3. Results

The physical characteristics for all participants are summarized in Table 1. All participants were physically active and do not have caffeine consumption habit.

**Table 1.** Participants characteristics for all participants. Data are expressed as mean ± SD.

Variables	
Age (years)	20.0 ± 1.7
Height (cm)	181.3 ± 6.7
Weight (kg)	74.0 ± 9.0
BMI (kg/m^2^)	22.5 ± 1.7
Physical activity (MET-min/w)	4787.5 ± 1067.4
Habitual caffeine intake (mg/day）	17.6 ± 6.0

**Figure 1.**
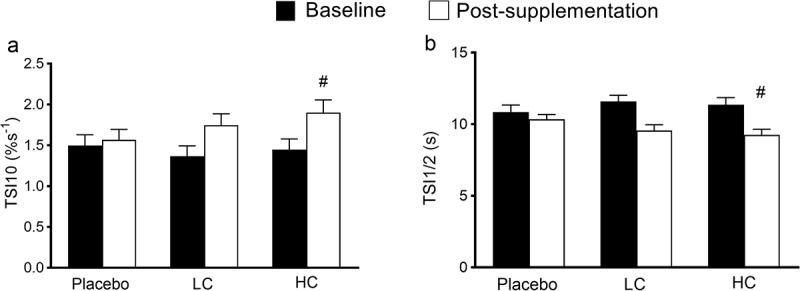
Near-infrared spectroscopy measured tissue saturation index 10s slope (TSI10) and TSI half time recovery (TSI ½) following tourniquet release before and after coffee consumption at rest. Placebo, decaf supplementation condition; LC, low caffeine content coffee supplementation at 3 mg/kg body weight condition; HC, high caffeine content coffee supplementation at 6 mg/kg body weight condition. #, significant difference compared to baseline.

**Figure 2.**
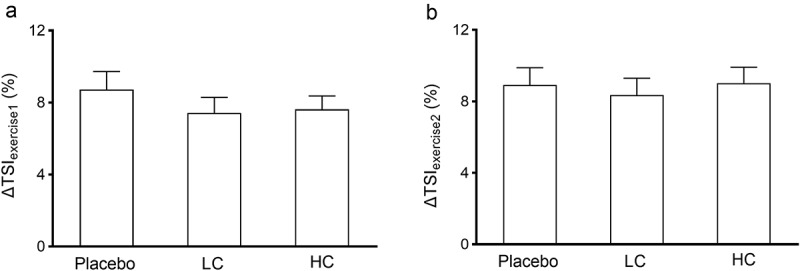
Near-infrared spectroscopy measured skeletal muscle microvascular oxygen extraction during exercise. Placebo, decaf supplementation condition; LC, low caffeine content coffee supplementation at 3 mg/kg body weight condition; HC, high caffeine content coffee supplementation at 6 mg/kg body weight condition. ΔTSI_exercise1_, the change in TSI from the first to the last common stage among three trials; ΔTSI_exercise2_, the TSI change from first to the last stage that the participant was able to complete for each individual trial.

For skeletal muscle reactivity measured at rest TSI10, it was revealed there was no difference in baseline measures among the three conditions (*p* = 0.705). After coffee ingestion, TSI10 was significantly higher for LC compared to placebo (*p* = 0.001), and was significantly higher for HC compared to placebo (*p* < 0.001), however no difference was found between LC and HC condition (*p* = 0.527). Furthermore, it was revealed that there was a significant interaction effect for TSI ½ (*p* = 0.008; ηp^2^ = 0.446), further analyses using one-way repeated ANOVA suggested that no difference was detected for baseline measures in TSI ½ among the three conditions (*p* = 0.372; ηp^2^ = 0.046); on the contrary, while no difference was found between placebo and LC conditions (*p* = 0.110), HC condition showed significant less TSI ½ compared to placebo (*p* = 0.005, Figure 1), indicating faster recovery. However, for microvascular oxygen extraction assessments, no difference was detected among the three conditions for ΔTSI_exercise1_ (*p* = 0.121; ηp^2^ = 0.105) or ΔTSI_exercise2_ (*p* = 0.355; ηp^2^ = 0.018, Figure 2).

There was an evident dose-dependent effect for W_peak_ (*p* < 0.001; ηp^2^ = 0.732). Participants at LC condition showed higher W_peak_ compared to placebo (268.1 ± 45.4 W vs. 246.7 ± 40.3 W; *p* < 0.001); similarly, participants at HC condition showed higher W_peak_ compared to LC (275.1 ± 46.1 W vs. 268.1 ± 45.4 W; *p* = 0.008) and placebo (*p* < 0.001). In addition, one-way repeated ANOVA test indicated that no differences exist among the three conditions for VO_2max_ (3.5 ± 0.5 L/min vs. 3.6 ± 0.6 L/min vs. 3.7 ± 0.5 L/min for placebo, LC and HC conditions, respectively; *p* = 0.093; ηp^2^ = 0.117).

## 4. Discussion

This study evaluated the acute effects of coffee intake with varying caffeine content, on skeletal muscle microvascular reactivity at rest and during incremental exercise. The primary finding was that coffee intake with high level of caffeine (6 mg/kg body weight) resulted in a significant enhancement in muscle microvascular reactivity at rest, which highlights the potential of caffeine as a potent modulator of vascular response. On the other hand, no discernable difference was observed among the three conditions for microvascular oxygen extraction during exercise. Furthermore, HC condition elicited greater W_peak_ compared to placebo and LC condition.

Although extensively studied regarding its ergogenic effects in exercise, the effects of coffee intake on peripheral microcirculation remain largely unknown. The finding of improvement in skeletal muscle reactivity at rest after HC coffee intake coincides with previous studies which indicate that skin microvascular function is elevated with acute coffee intake [4,25]. Our finding is crucial, as it is well established that the microvascular network plays a pivotal role in physical exertion and post-exercise recovery [26]. The enhanced microvascular reactivity could suggest more efficient oxygen and nutrients delivery into the skeletal muscle tissue, thus provide basis for better exercise performance and facilitate the recovery process. Importantly, this change in microvascular function could also be linked to increased removal of metabolic byproducts post-exercise, leading to a faster recovery process. This is substantiated by one report which indicates that caffeine intake results in alleviation in delayed onset muscle soreness and performance reduction as induced by muscle damage in elite collegiate athletes [27].

The mechanisms through which coffee exerts its impact on skeletal muscle microcirculation could be complicated, and among its many constitutes that may have ergogenic effects, caffeine is likely to be the primary contributor. The primary mechanism may be related to its effect on the endothelial function, which has been studied extensively. Caffeine has been shown to upregulate nitric oxide (NO) release, thereby leading to improved endothelial function and hence vasodilation. It was demonstrated that intake of caffeinated coffee leads to stronger forearm skin vasodilation response during iontophoresis of acetylcholine compared to decaffeinated coffee, suggesting an endothelium-dependent vasodilation mechanism, while the endothelium-independent vasodilation was not affected [4]. This suggests that the caffeine exerts its impact on microvasculature through the modulation of NO production. Additionally, caffeine is also known to influence the sympathetic nervous system by stimulating catecholamine release [28]. Apart from affecting heart rate and blood pressure, it’s possible that changes in catecholamine levels could also have an effect on the microvascular function [29]. Specifically, because the skeletal muscle vasculature contains predominantly β2-adrenergic receptors, the binding of epinephrine and receptors within the skeletal muscle is likely to result in vasodilation, leading to the increased microvascular reactivity observed within the current study. Furthermore, it was suggested that caffeine can affect the calcium mobilization within the smooth muscle cells. Such an action results in alterations in the intracellular calcium levels which lead to changes in vascular tone and blood flow [30]. Taken together, it’s clear that caffeine content within the coffee intake could affect skeletal muscle microvascular reactivity through various mechanisms, and further investigation is needed to elucidate their complexed interplay. On the other hand, it should be noted that coffee contains many other constituents, such as polyphenols and diterpenes, which may also contribute to its effects on the microcirculation and warrant further exploration.

The finding of no discernable difference among three conditions for skeletal muscle microvascular oxygen extraction during exercise is contradictory to our hypothesis, and this is particularly interesting given a clear improvement in W_peak_. Our finding is similar to a recent study which indicate that during all-out exercise, caffeine intake offered no benefits for muscle oxygen saturation, and its ergogenic effects is mainly due to increased glycolytic metabolism [31]. Previous studies have demonstrated that coffee can significantly enhance muscular endurance [32]. However, findings from this study suggest that such effect is not attributed to the direct alterations in skeletal muscle oxygenation. The improved maximal aerobic power could be the result of increased muscle contractility [33] and decreased perception of exertion [34]. Future research is needed to comprehensively evaluate the mechanisms through which coffee influences exercise performance. In addition, whether a higher dose (9 mg/kg) could influence muscle dynamic oxygen extraction during exercise is unknown and is worth further exploration.

Our study provides evidence that the acute intake of coffee could significantly enhance skeletal muscle microvascular reactivity at rest and improve exercise capacity. Findings of this study highlighted the ergogenic role of coffee, and support the use of coffee as a valuable supplement for exercise performance and post-exercise recovery. While not directly investigated in the current study, the vascular effect observed here could suggest a potential dietary therapeutic effect for coffee in people with compromised vascular function, although clearly more research is needed to confirm this notion. Further, the findings regarding W_peak_ were similar to previous studies and suggest the role of caffeine as a modulator for endurance sports where circulation and oxygen utilization are crucial.

Limitations of this study must be addressed. The first limitation is the study sample. This study employed solely young, healthy, physically active male individuals. Therefore, the results of this study should not be carried to other populations without specifically designed studies. Secondly, the highest amount of caffeine content in the coffee used in the current study was 6 mg/kg body mass. Whether a higher dose (9 mg/kg body mass) could induce the dose-dependent increase in skeletal muscle microvascular reactivity at rest or will higher dose potentially hamper such response, is not addressed in our study and warrants further investigation. Thirdly, this study performed a single post-intake measure on microvascular function in the muscle. Therefore, it’s unclear how long such effect may last, and whether chronic coffee intake could lead to favorable changes is still unknown and should be further explored. In addition, it should be noted that coffee has many constitutes other than caffeine that may potentially influence the human physiological system. Future studies with similar purposes should consider comparing the effects of coffee ingestion to pure caffeine ingestion to elucidate the specific contributions of other coffee constituents. Such comparison would help determine whether other components in coffee also play a significant role in the observed physiological effects.

## 5. Conclusions

In conclusion, in this study we found that acute coffee intake with high caffeine level (6 mg/kg body weight) significantly enhanced skeletal muscle reactivity at rest. Maximal aerobic power was also improved, indicating the ergogenic potential of coffee. However, the improvement of exercise performance with coffee intake is not accompanied by alterations in muscle microvascular oxygen extraction.

## Data Availability

Data of this study will be available upon request to the corresponding author on reasonable noncommercial request.
